# Network Pharmacology and Experimental Validation to Reveal the Pharmacological Mechanisms of Chongcaoyishen Decoction Against Chronic Kidney Disease

**DOI:** 10.3389/fmolb.2022.847812

**Published:** 2022-03-31

**Authors:** Zhenliang Fan, Jingjing Chen, Qiaorui Yang, Jiabei He

**Affiliations:** ^1^ Nephrology Department, The First Affiliated Hospital of Zhejiang Chinese Medical University, Hangzhou, China; ^2^ Department of Rheumatology and Immunology, The First Hospital Affiliated to Army Medical University, Chongqing, China; ^3^ Graduate School, Heilongjiang University of Chinese Medicine, Harbin, China; ^4^ Department of Oncology Radiotherapy, Affiliated Zhongshan Hospital to Dalian University, Liaoning, China

**Keywords:** Chongcaoyishen decoction, chronic kidney disease, oxidative stress injury, autophagy, mitochondrial injury, tubulointerstitial fibrosis

## Abstract

**Objective:** To explore the pharmacological mechanisms of Chongcaoyishen decoction (CCYSD) against chronic kidney disease (CKD) *via* network pharmacology analysis combined with experimental validation.

**Methods:** The bioactive components and potential regulatory targets of CCYSD were extracted from the TCMSP database, and the putative CKD-related target proteins were collected from the GeneCards and OMIM database. We matched the active ingredients with gene targets and conducted regulatory networks through Perl5 and R 3.6.1. The network visualization analysis was performed by Cytoscape 3.7.1, which contains ClueGO plug-in for GO and KEGG analysis. *In vivo* experiments were performed on 40 male SD rats, which were randomly divided into the control group (*n* = 10), sham group (*n* = 10), UUO group (*n* = 10), and CCYSD group (*n* = 10). A tubulointerstitial fibrosis model was constructed by unilateral ureteral obstruction through surgery and treated for seven consecutive days with CCYSD (0.00657 g/g/d). At the end of treatment, the rats were euthanized and the serum and kidney were collected for further detection.

**Results:** In total, 53 chemical compounds from CCYSD were identified and 12,348 CKD-related targets were collected from the OMIM and GeneCards. A total of 130 shared targets of CCYSD and CKD were acquired by Venn diagram analysis. Functional enrichment analysis suggested that CCYSD might exert its pharmacological effects in multiple biological processes, including oxidative stress, apoptosis, inflammatory response, autophagy, and fiber synthesis, and the potential targets might be associated with JAK-STAT and PI3K-AKT, as well as other signaling pathways. The results of the experiments revealed that the oxidative stress in the UUO group was significantly higher than that in normal state and was accompanied by severe tubulointerstitial fibrosis (TIF), which could be effectively reversed by CCYSD (*p* < 0.05). Meanwhile, aggravated mitochondrial injury and autophagy was observed in the epithelial cells of the renal tubule in the UUO group, compared to the normal ones (*p* < 0.05), while the intervention of CCYSD could further activate the autophagy and reduce the mitochondrial injury (*p* < 0.05).

**Conclusion:** We provide an integrative network pharmacology approach combined with *in vivo* experiments to explore the underlying mechanisms governing the CCYSD treatment of CKD, which indicates that the relationship between CCYSD and CKD is related to its activation of autophagy, promotion of mitochondrial degradation, and reduction of tissue oxidative stress injury, promoting the explanation and understanding of the biological mechanism of CCYSD in the treatment of CKD.

## Introduction

Chronic kidney disease (CKD) mainly refers to the irreversible structural and/or functional impairment of the kidney resulted from multiple causes for more than 3 months. With the change of people’s lifestyle, CKD has become a chronic disease seriously affecting human health along with chronic diseases, such as diabetes and hypertension (Webster et al., 2017). The increasing prevalence of CKD has been accompanied by the increasing healthcare costs. According to incomplete statistics, the annual cost of CKD patients in the world is up to five billion dollars, and nearly one million CKD patients die because they cannot afford the high cost of treatment (Couser et al., 2011; Djudjaj and Boor, 2019).

However, the treatment of chronic kidney disease (CKD) is not a breakthrough which deserves celebrating at present. The mainstay of therapy for CKD contains angiotensin-converting enzyme inhibitor and angiotensin II inhibitor that can alleviate glomerular “three highs” status (Aggarwal and Singh, 2020) by means of controlling the blood pressure and blood glucose, correcting metabolic acidosis with sodium bicarbonate, restricting protein intake, and taking alpha-keto acid. However, these conventional treatment measures are mainly symptomatic treatment and the suboptimal therapy delaying renal function decline, and treating related complications does not always work effectively because it is hard to grasp the key of pathological changes and pathogenesis of CKD (Collins et al., 2012; National Kidney Foundatio, 2012; Humphreys, 2018). Therefore, it has been a hot issue in relevant research fields to find out drugs that can effectively delay the deterioration of renal function and improve the prognosis by targeting the core pathological changes in the progression of CKD (Gewin, 2018).

With the continuous promotion of traditional Chinese medicine (TCM) in the clinical treatment of CKD, its efficacy in delaying the deterioration of renal function and improving the prognosis of patients has gradually been approved by clinicians and patients. For this reason, many emerging studies in recent years have gradually revealed the mechanism of action and intervention targets of TCM in the treatment of CKD. According to the current research results, TCM compound has the advantage of multiple components and multiple targets in the treatment of CKD, which is incomparable to Western drugs with single chemical components, and can simultaneously intervene multiple targets closely related to the progress of CKD.

Chongcaoyishen decoction (CCYSD) has been applied in clinical practice for many years. Based on the pathological characteristics of patients with CKD, it emphasizes that the treatment should focus on “supplementing and removing the deficiency and combining reinforcement with elimination.” Previous studies have fully confirmed that CCYSD can effectively delay renal deterioration in patients with CKD, relieve clinical symptoms, and improve the prognosis of patients (Mo et al., 2019b; Ziyang, 2019; Ma et al., 2020). Although the researchers have carried out many basic studies before, we still cannot figure out a definite explanation to the specific target and exact mechanism of CCYSD in the treatment of chronic kidney disease. In this study, we used network pharmacology to explore the bioactive ingredients in CCYSD and its mechanism of action in treating CKD. Subsequently, experimental verification was carried out on the results of the network pharmacology study to further explore the specific mechanisms of CCYSD in treating CKD.

## Data and Methods

### Network Pharmacology Analysis

#### Data Sources

In this study, the bioactive ingredients of Chongcaoyishen decoction and the possible intervention targets of CCYSD were screened from the Traditional Chinese Medicine Systems Pharmacology Database and Analysis Platform (TCMSP). The targets related to chronic kidney disease were extracted from GeneCards and OMIM databases, and ClueGO plug-in from Cytoscape 3.7.1 was used for Gene Ontology (GO) and Kyoto Encyclopedia of Genes and Genomes (KEGG) enrichment analysis in order to analyze the biological functions and signaling pathways involved in the drug regulatory network.

#### Screening of Active Components and Targets of Chongcaoyishen Decoction

The bioactive ingredients of *Cordyceps sinensis*, *Astragalus membranaceus*, leeches, rhubarb in wine, cardamom, and *Sergium sergii* were searched in the TCMSP database, and the bioactive ingredients and their corresponding targets were reserved with oral bioavailability (OB) ≥30% and drug-like property (DL) ≥0.18 as screening conditions.

#### CKD Target Screening

Taking “chronic kidney diseases” as the keyword, we surveyed the related targets of CKD from GeneCards (https://www.genecards.org/) and retrieved 12,183 genes associated with CKD. A total of 202 related gene targets were retrieved from Online Mendelian Inheritance in Man (OMIM, https://omim.org/). After removing the duplicates from GeneCards and OMIM databases, we obtained 12,348 non-repeating gene targets.

#### Construction of a Drug Regulatory Network and Functional Enrichment Analysis

Based on the collected data, we constructed the drug regulatory network using Perl5 and R 3.6.1 to showcase the correlation between the common targets of active ingredients in CCYSD and potential targets for CKD.

The common targets of active ingredients in CCYSD and potential targets for CKD were collected and input into Cytoscape 3.7.1 to construct a CKD–CCYSD–ingredients–target interaction network, which showcases the abundance of bioactive constituents from CCYSD exerts therapeutic effects on CKD through multiple gene targets.

Meanwhile, the plug-in from Cytoscape 3.7.1, ClueGO, was used to perform visual analysis of KEGG and GO functional enrichment. The KEGG and GO pathway analyses were screened for kappa >0.74 and *p* < 0.01. The top 30 items of GO analysis and the top 30 items of KEGG analysis were mapped as bar plots and bubble plots, and the positions of relevant nodes were adjusted, aiming to obtain a clearer network graph.

### Experimental Research

#### Animals and Experimental Groups

A total of 40 SPF Sprague Dawley (SD) rats (180–220 g) were supplied by the Experimental Animal Center of Heilongjiang University of Chinese Medicine [SCXK (Black) 2017-014]. All these rats were housed in an environmentally controlled room (22 ± 2°C, humidity 60 ± 10%, 12 h/12 h light/dark cycle) with free access to water and lab chow.

According to the random number table, all the animals were randomly and equally divided into four groups: the control group, sham group, UUO group, and CCYSD group (*n* = 10 in each group).

#### Main Reagents

ELISA 96-well kit: superoxide dismutase (SOD, Solebo BC0170), reduced glutathione (GSH, Solebo BC1170), and malondialdehyde (MDA, Solebo BC0020). Western blot primary antibody: GAPDH (Cymofei MA5-15738), α-SMA (Cymofei MA1-06110), COL-III (CSI007-01-02), and LC3B (PA1-46286).

#### TIF Modeling and Drug Administration

Unilateral ureteral obstruction (UUO) was established by the surgical method. In brief, rats were anesthetized with pentobarbital, and then, the left renal ureter was separated from the abdomen. After ureter ligation and severance, the muscles and skin were sutured layer by layer to allow the rats to recover. The rats in the UUO group and CCYSD group received the UUO model, while the rats in the sham group received a similar surgical approach without ligation or severance, and the rats in the control group did not undergo any treatment.

The day after the surgery, the rats in the CCYSD group were administered intragastrically with CCYSD at a dose of 0.00657 g/g/d once a day for seven consecutive days, which is equal to the decoction with a dose of 0.657 g/ml (Jie, 2015; Fan et al., 2019; Mo et al., 2019a). The rats in the sham group and UUO group were given the same dosage of normal saline (2 ml). The control group received nothing. Seven days after the surgery, all rats were euthanized, and the blood and tissues were collected for further analysis.

#### Sample Collection

2 h after the last administration, 5 ml of inferior venous blood was collected from anesthetized rats and centrifuged at 3,000 r/min for 15 min at 4°C to separate the serum. The left kidney was separated into three parts and stored in a 2.5% glutaraldehyde electron microscopy fixative solution, 10% neutral formalin fixative solution, and liquid nitrogen, respectively.

#### Enzyme Linked Immunosorbent Assay

The serum oxidative stress-related markers were detected by ELISA: the spectrophotometer was preheated for 30 min and zeroed with distilled water. Superoxide dismutase (SOD, Solebo BC0170), reduced glutathione (GSH, Solebo BC1170), and malondialdehyde (MDA, Solebo BC0020) were tested according to the corresponding instructions.

#### Histological Analysis

Pathological observation: the renal tissues were fixed by formalin, embedded by paraffin, and sectioned at 3 μm. H&E staining was performed with hematoxylin and eosin staining after gradient elution. Then, the specimen was washed with running water again, dehydrated by graded ethanol, and vitrified by dimethylbenzene, and the neutral resin was used for sealing. Weigert ferric hematoxylin staining solution, Masson cyanating solution, and lichunred fuchsin staining solution were used for Masson staining in sequence. Three fields were randomly selected for each section, and the proportion of interstitial fibrosis area was calculated with ImageJ software. Electron microscopy: the renal tissue was fixed with glutaraldehyde first and with 2% osmium tetroxide solution later. Then, the samples were dehydrated by epoxy propylene, and the epoxy resin 828 was substituted for it at 35 and 45°C for 12 h, respectively.

The specimen was heated at 60°C for 48 h before repairing and cutting into semi-thin slices. Methylene blue was applied to dye and locate the specimens, which were then cut into ultra-thin slices. Uranyl acetate and lead citrate were used for double staining in order to observe the slices under transmission electron microscopy (JED1400PLUS).

#### Western Blot

Immunoblotting was performed using standard protocols with frozen tissues which were ground in liquid nitrogen. RIPA lysis buffer, benzonase nuclease, protease, and phosphatase inhibitors, used in the study, were added to lysate at room temperature, and the supernatant was extracted by centrifugation at 13,000 r/min for 10 min. Total protein concentration was determined by the BCA method. After 150 V electrophoresis, the membrane was incubated with primary antibody and then secondary antibody. Immunoblots were treated with a chemiluminescence detection system followed by the exposure to Hyperfilm ECL.

#### Statistical Analysis

All data collected in this study were analyzed using IBM-SPSS Statistics 26.0 software, and the continuous measurement data were expressed as the mean ± standard deviation ( $\bar{x}$ ± s). The data, which coincide with normal distribution and satisfy homogeneity variance, between groups were compared through one-way ANOVA (Table 1) and that between groups with the Tukey method. Otherwise, significant differences were analyzed by the Kruskal–Wallis test and tested with the Kruskal–Wallis test and Mann–Whitney U rank-sum test (Figures 7, 8B). *p* < 0.05 was considered statistically significant.

**TABLE 1:** Serum oxidative stress levels of rats in each group ( $\bar{X}\pm S$ ).

	Malondialdehyde (nmol/mL)	Superoxide dismutase (U/mL)	Reduced glutathione (μg/mL)
Control group	7.90 ± 0.80	263.89 ± 35.01	645.42 ± 84.98
Sham group	7.38 ± 0.49	244.25 ± 24.51	655.68 ± 82.92
UUO group	8.68 ± 0.42*	193.80 ± 32.52*	406.60 ± 55.72*
CCYSD group	7.39 ± 0.45#	265.18 ± 28.85#	589.18 ± 65.45#

*Compared with the sham group, *p* < 0.05; #compared with the UUO group, *p* < 0.05.

## Results

Active ingredients in CCYSD: by retrieving the TCMSP database, a total of 53 kinds of non-repeating bioactive ingredients of CCYSD were selected, including organic acids, lipids, biophenols, sterols, flavonoids, and other main compounds, for example, catechuic acid, arachidonic acid, procyanidin B-5,3'-O-gallic acid, rhein acid, allyl peroxide, biphenyl diester, toralactone, dehydrodiiso-eugenol, kaempferol, β-sitosterol, cerevisterol, ergosterol peroxide, calycosin, isoflavone, and foxglove flavonoid (Table 2).

**TABLE 2:** Biological active ingredients in CCYSD.

Mol ID	Molecule Name	OB (%)	Caco-2^a^	DL
MOL000096	(-)-Catechin	49.68	−0.03	0.24
MOL000228	(2R)-7-hydroxy-5-methoxy-2-phenylchroman-4-one	55.23	0.87	0.2
MOL000438	(3R)-3-(2-hydroxy-3,4-dimethoxyphenyl) chroman-7-ol	67.67	0.96	0.26
MOL000033	(3S,8S,9S,10R,13R,14S,17R)-10,13-dimethyl-17-[(2R,5S)-5-propan-2-yloctan-2-yl] c-2,3,4,7,8,9,11,12,14,15,16,17-dodecahydro-1H-cyclopenta [a] phenanthren-3-ol	36.23	1.45	0.78
MOL000224	(4E,6E)-1,7-bis (3,4-dihydroxyphenyl) hepta-4,6-dien-3-one	33.06	0.29	0.31
MOL000380	(6aR,11aR)-9,10-dimethoxy-6a,11a-dihydro-6H-benzofurano [3,2-c] chromen-3-ol	64.26	0.93	0.42
MOL000442	1,7-Dihydroxy-3,9-dimethoxy pterocarpene	39.05	0.89	0.48
MOL000235	1,7-Diphenyl-3,5-dihydroxy-1-heptene	49.01	0.61	0.18
MOL000238	1,7-Diphenyl-5-hydroxy-6-hepten-3-one	32.65	0.8	0.18
MOL000371	3,9-Di-O-methylnissolin	53.74	1.18	0.48
MOL000260	5-[(2R,3R)-7-methoxy-3-methyl-5-[(E)-prop-1-enyl]-2,3-dihydrobenzofuran-2-yl]-1,3-benzodioxole	65.55	1.27	0.4
MOL000374	5'-Hydroxyiso-muronulatol-2',5'-di-O-glucoside	41.72	−2.47	0.69
MOL000242	7-O-Methyleriodictyol	56.56	0.46	0.27
MOL000378	7-O-Methylisomucronulatol	74.69	1.08	0.3
MOL000379	9,10-Dimethoxypterocarpan-3-O-β-D-glucoside	36.74	−0.63	0.92
MOL000471	Aloe emodin	83.38	−0.12	0.24
MOL000243	Alpinolide peroxide	87.67	0.51	0.19
MOL001439	Arachidonic acid	45.57	1.2	0.2
MOL000358	Beta-sitosterol	36.91	1.32	0.75
MOL000387	Bifendate	31.1	0.15	0.67
MOL000417	Calycosin	47.75	0.52	0.24
MOL008998	Cerevisterol	39.52	0.35	0.77
MOL008999	Cholesteryl palmitate	31.05	1.45	0.45
MOL000953	CLR	37.87	1.43	0.68
MOL000274	Cordycepin	45.37	0.79	0.87
MOL002297	Daucosterol_qt	35.89	1.35	0.7
MOL000258	Dehydrodiisoeugenol	56.84	1.19	0.29
MOL002288	Emodin-1-O-beta-D-glucopyranoside	44.81	−1.12	0.8
MOL002235	EUPATIN	50.8	0.53	0.41
MOL000433	FA	68.96	−1.5	0.71
MOL000392	Formononetin	69.67	0.78	0.21
MOL000554	Gallic acid-3-O-(6'-O-galloyl)-glucoside	30.25	−1.96	0.67
MOL000296	Hederagenin	36.91	1.32	0.75
MOL000398	Isoflavanone	109.99	0.53	0.3
MOL000439	Isomucronulatol-7,2'-di-O-glucosiole	49.28	−2.22	0.62
MOL000354	Isorhamnetin	49.6	0.31	0.31
MOL000239	Jaranol	50.83	0.61	0.29
MOL000422	Kaempferol	41.88	0.26	0.24
MOL001645	Linoleyl acetate	42.1	1.36	0.2
MOL000006	Luteolin	36.16	0.19	0.25
MOL000211	Mairin	55.38	0.73	0.78
MOL002251	Mutatochrome	48.64	1.97	0.61
MOL002303	Palmidin A	32.45	−0.36	0.65
MOL011169	Peroxyergosterol	44.39	0.86	0.82
MOL002259	Physciondiglucoside	41.65	−2.64	0.63
MOL000230	Pinocembrin	57.56	0.38	0.2
MOL002260	Procyanidin B-5,3'-O-gallate	31.99	−1.61	0.32
MOL000098	Quercetin	46.43	0.05	0.28
MOL002268	Rhein	47.07	−0.2	0.28
MOL002293	Sennoside D_qt	61.06	−0.7	0.61
MOL002276	Sennoside E_qt	50.69	−0.74	0.61
MOL002280	Torachrysone-8-O-beta-D-(6'-oxayl)-glucoside	43.02	−1.23	0.74
MOL002281	Toralactone	46.46	0.86	0.24

a Caco-2: permeability.

### Potential Target Prediction of CCYSD

**FIGURE 1:**
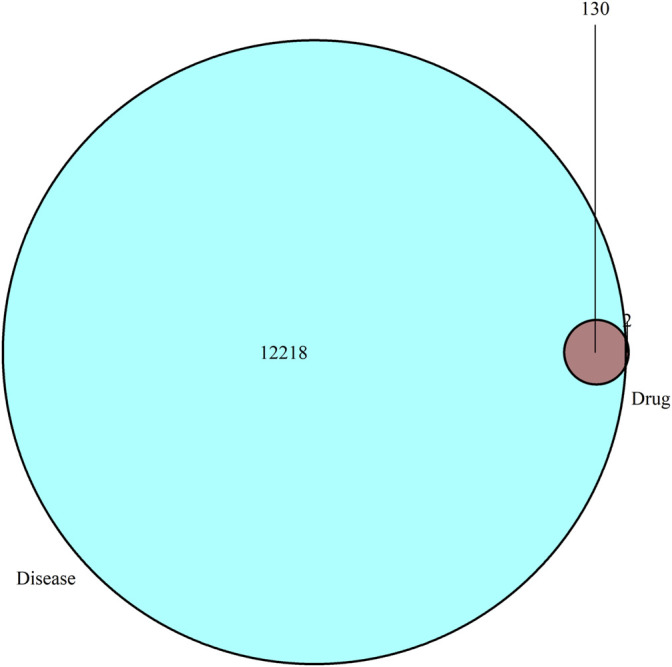
Venn diagram summarizing the intersection targets of CCYSD and CKD.

To identify the intersection of CCYSD ingredients and CKD targets, a Venn diagram analysis was carried out. A total of 132 potential targets were identified, which matched with the related targets of 53 active ingredients. 12,348 non-repeating targets closely related to CKD were screened from GeneCards and OMIM databases. As shown in Figure 1, 130 intersecting targets were obtained after matching.

### Regulatory Network Construction and Key Targets

**FIGURE 2:**
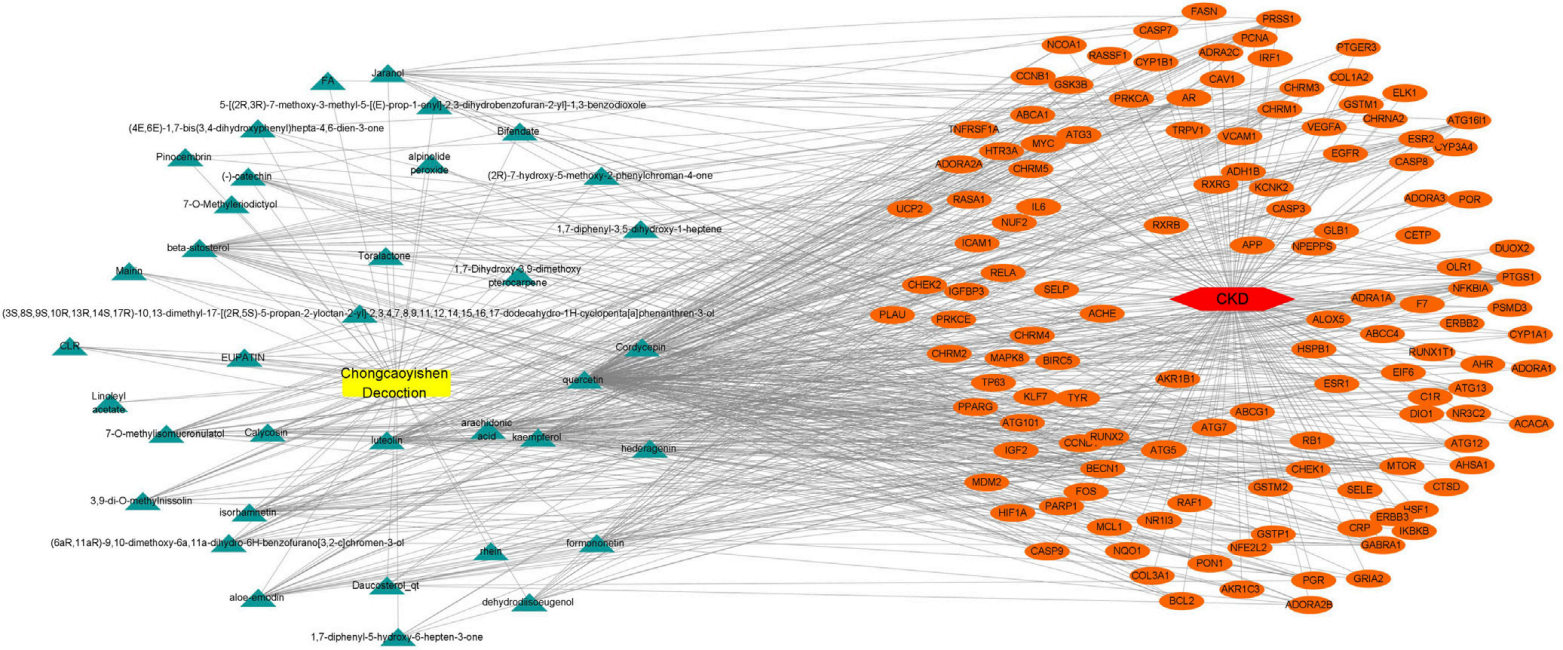
Regulatory network of CCYSD in the treatment of CKD.

To identify the core proteins of CCYSD intervention for CKD, a regulatory network was constructed by matching the ingredients of CCYSD with CKD targets and lines were drawn between them. At the same time, the importance of components and targets was evaluated according to the degree of connection between the components and targets in the regulatory network. As shown in Figure 2, quercetin, foxglove flavonoids, 7-O-methylthiamine, isorhamnetin, catechuic acid, aloe-emodin, kumatakenin, and cordycepin are at key positions in the regulatory network of CCYSD, which are closely related to multiple gene targets. Among the gene targets, PRSS1, CHRM3, ATG16l1, AR, PTGS1, MTOR, NFKBIA, ALOX5, ESR1, CRP, GABRA1, ADORA2B, COL3A1, ATG101, HIF1A, CASP9, ADORA2A, MAPK8, ATG 3. ATG5, ATG7, BECN1, ESR1, IL6, BCL2, and other gene targets are important in the regulatory network, indicating that these genes are involved in the occurrence and development of CKD and are regulated by CCYSD in different degrees, which may be central targets for the therapeutic effect of CCYSD.

### Enrichment Analysis of GO and KEGG

**FIGURE 3:**
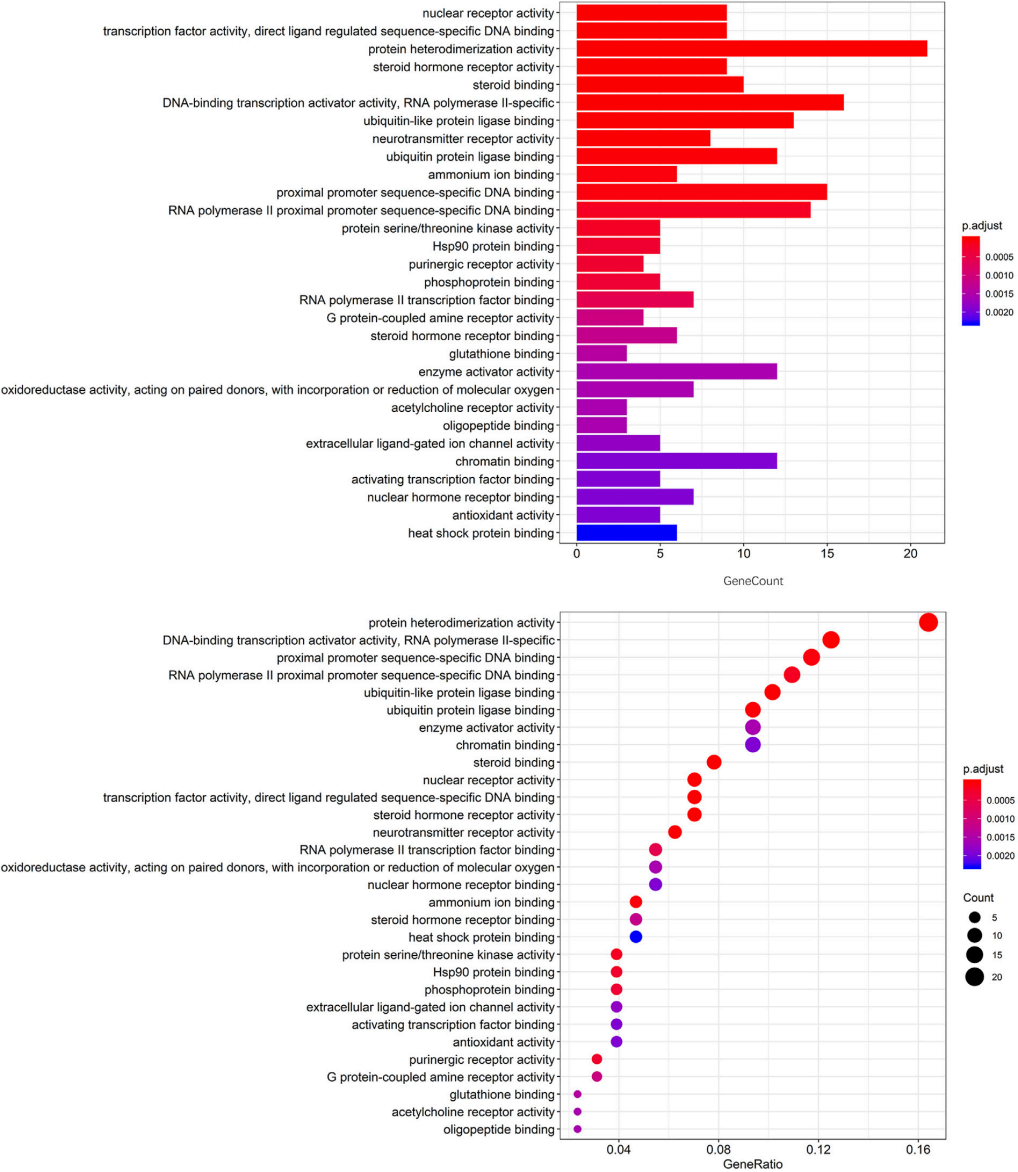
The top 30 of GO enrichment analysis.

**FIGURE 4:**
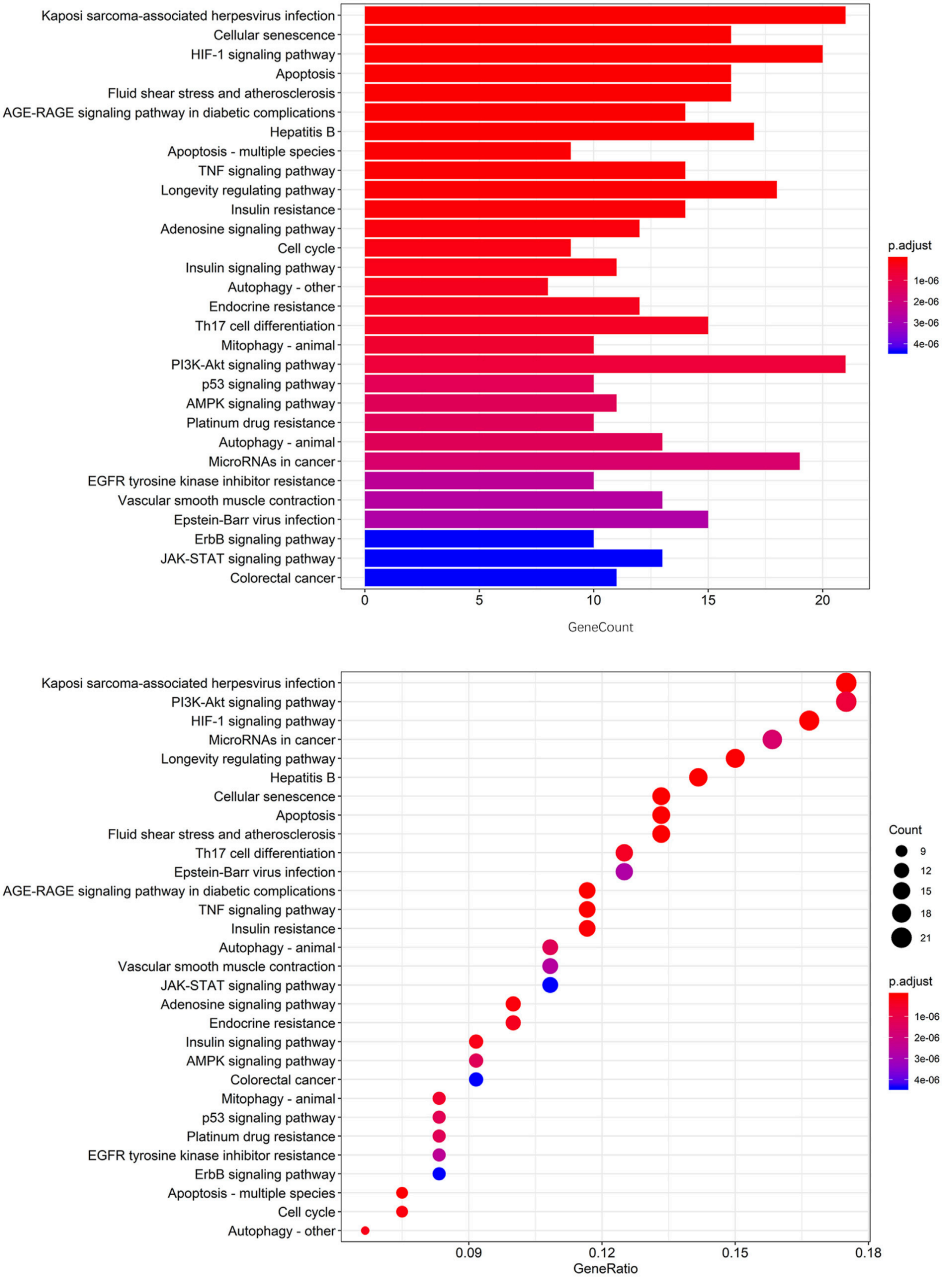
The top 30 signaling pathways from KEGG analysis.

To further explore the potential signaling pathways regulated by CCYSD, the functional enrichment analyses of GO and KEGG were carried out based on the potential targets of CCYSD *via* ClueGO plug-in from Cytoscape 3.7.1. Filtering criteria was set as *p* value cutoff = 0.05 and *q* value cutoff = 0.05. Finally, 114 biological progresses were obtained by GO functional enrichment analysis, and 112 related cell signaling pathways were obtained by KEGG pathway enrichment analysis (Figures 3, 4).

**FIGURE 5:**
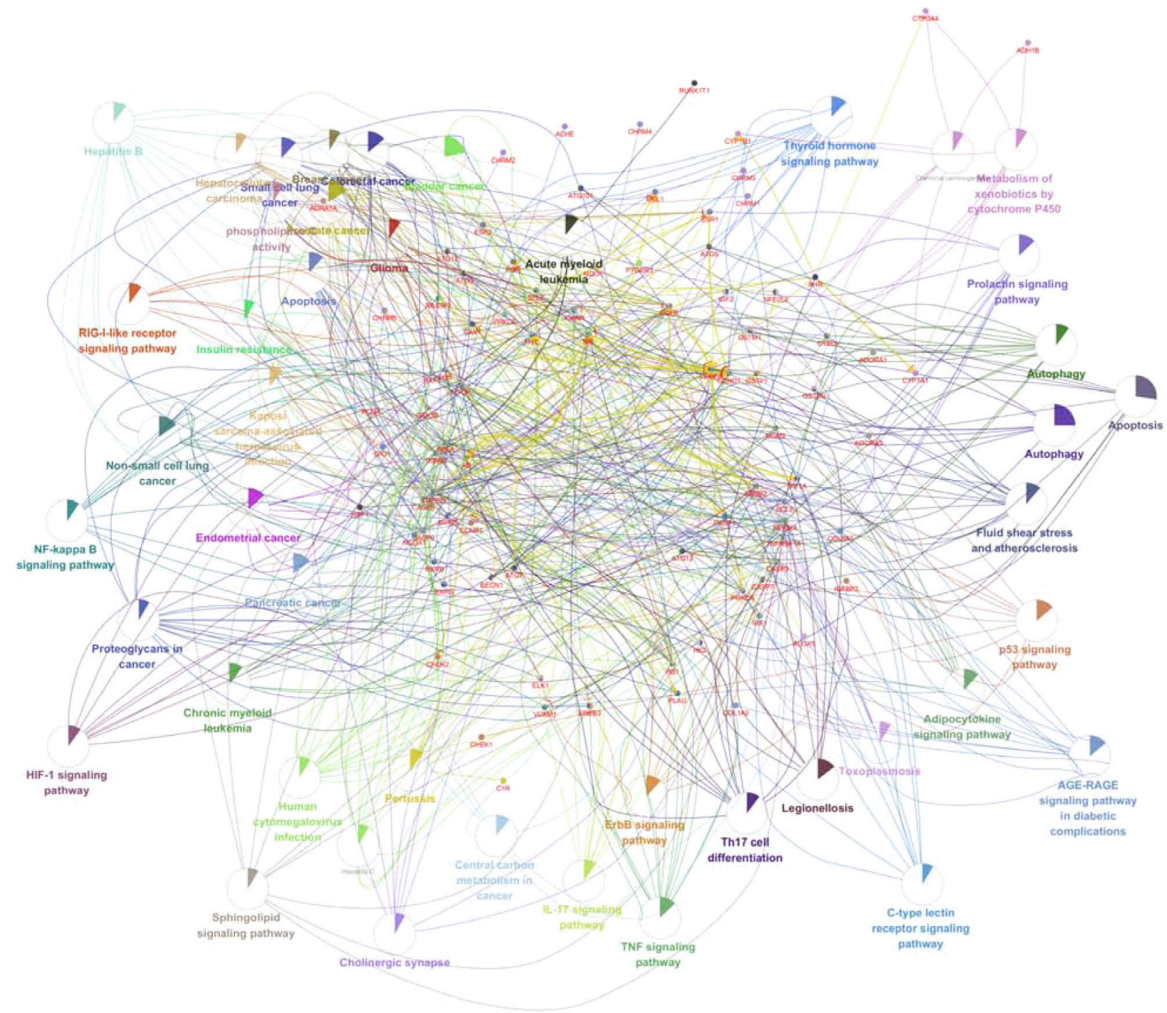
Mechanism network of CCYSD in the treatment of CKD.

Subsequently, 45 signaling pathways and functional activities with kappa >0.74 and *p* < 0.01 were labeled in the network by analyzing kappa statistics (Figure 5). As showcased in Figure 5, the pharmacological mechanisms of CCYSD in the treatment of chronic kidney disease may be involved with autophagy, apoptosis, the p53 signaling pathway, Th17-cell differentiation, adipocytokine signaling, C-type lectin receptor signaling, the ErbB signaling pathway, the TNF signaling pathway, the IL-17 signaling pathway, cholinergic protrusion signaling, the sheath ester signaling pathway, the NF-κB signaling pathway and HIF-1 signaling pathway, the RIG-1-like receptor signaling pathway, the phosphokinase C signaling pathway, and the cytochrome P450 system phagocytic metabolism of heterologous substances and other mechanisms.

Both GO and KEGG enrichment analysis suggested that the therapeutic effect of CCYSD on CKD was mostly related to the regulation of oxidative stress injury, inflammatory response, apoptosis, and autophagy in renal tissues. Further exploration of the abovementioned mechanisms in treating CKD and delaying renal tubulointerstitial fibrosis would be conducted in the subsequent experiments *in vivo*.

### Pathological Changes of Renal Tissue

**FIGURE 6:**
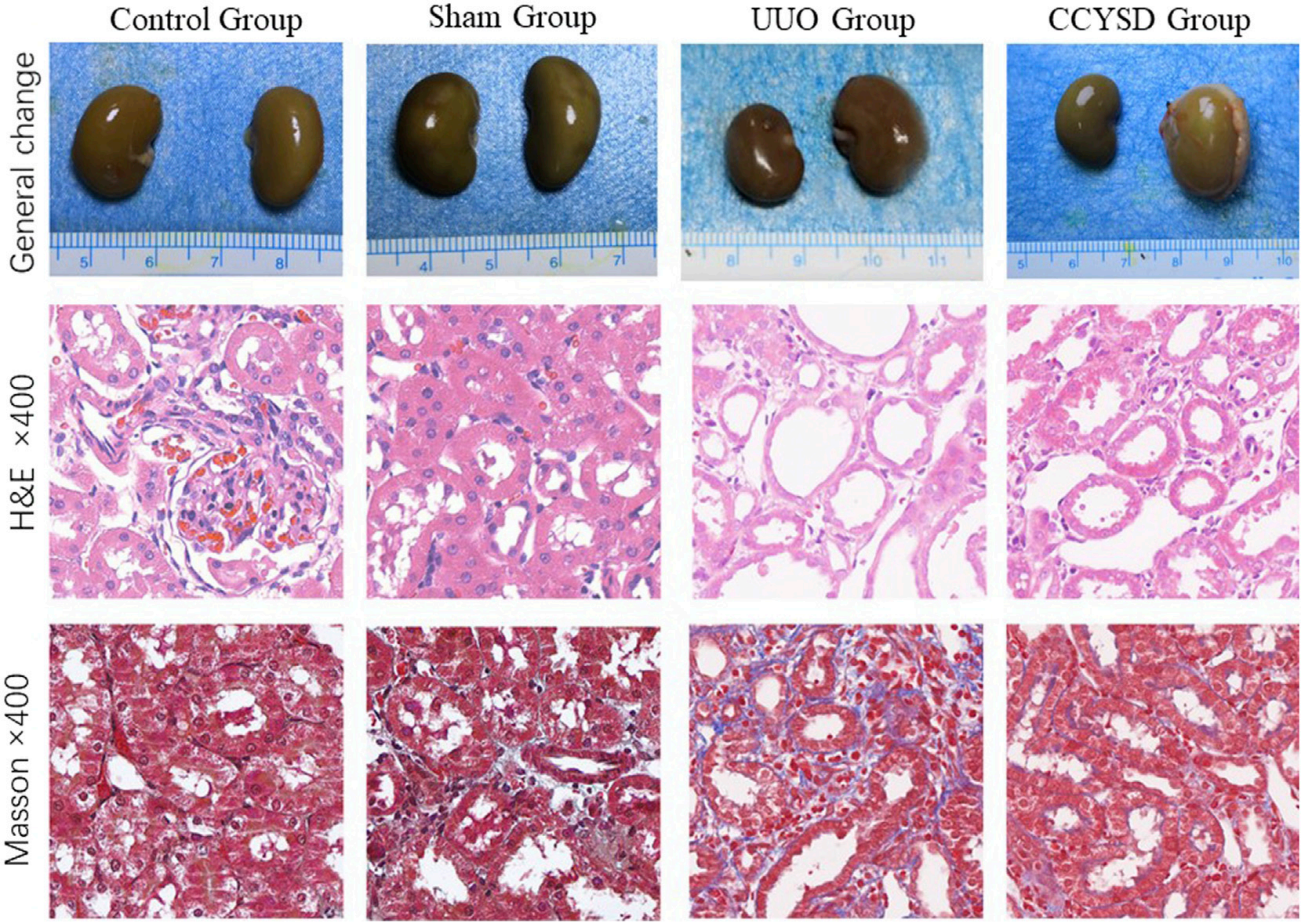
Pathological changes of renal tissue.

To further verify the key pharmacological mechanism of CCYSD in the treatment of CKD as predicted previously, UUO rats were constructed and administered intragastrically with CCYSD *in vivo*. According to Figure 6, the obstructed kidney tissues of UUO animals were significantly larger than that of the normal ones. Compared with the control group, the histological changes of the renal interstitium were examined by H&E staining, which exhibited the widened renal interstitium, dilated renal tubule, and a wide range of exfoliation in the brush border of renal tubular epithelial cells in the UUO group. More infiltration of inflammatory cells and interstitial hemorrhage were also seen in some fields. The results of Masson staining showed increased extracellular matrix and fibrous proliferation in the renal interstitium. Although obvious tubule damage and renal interstitial fibrosis were also observed in the CCYSD group, these pathological changes mentioned above can be reversed by CCYSD to some extent.

### Degree of Renal Tissue Fibrosis

**FIGURE 7:**
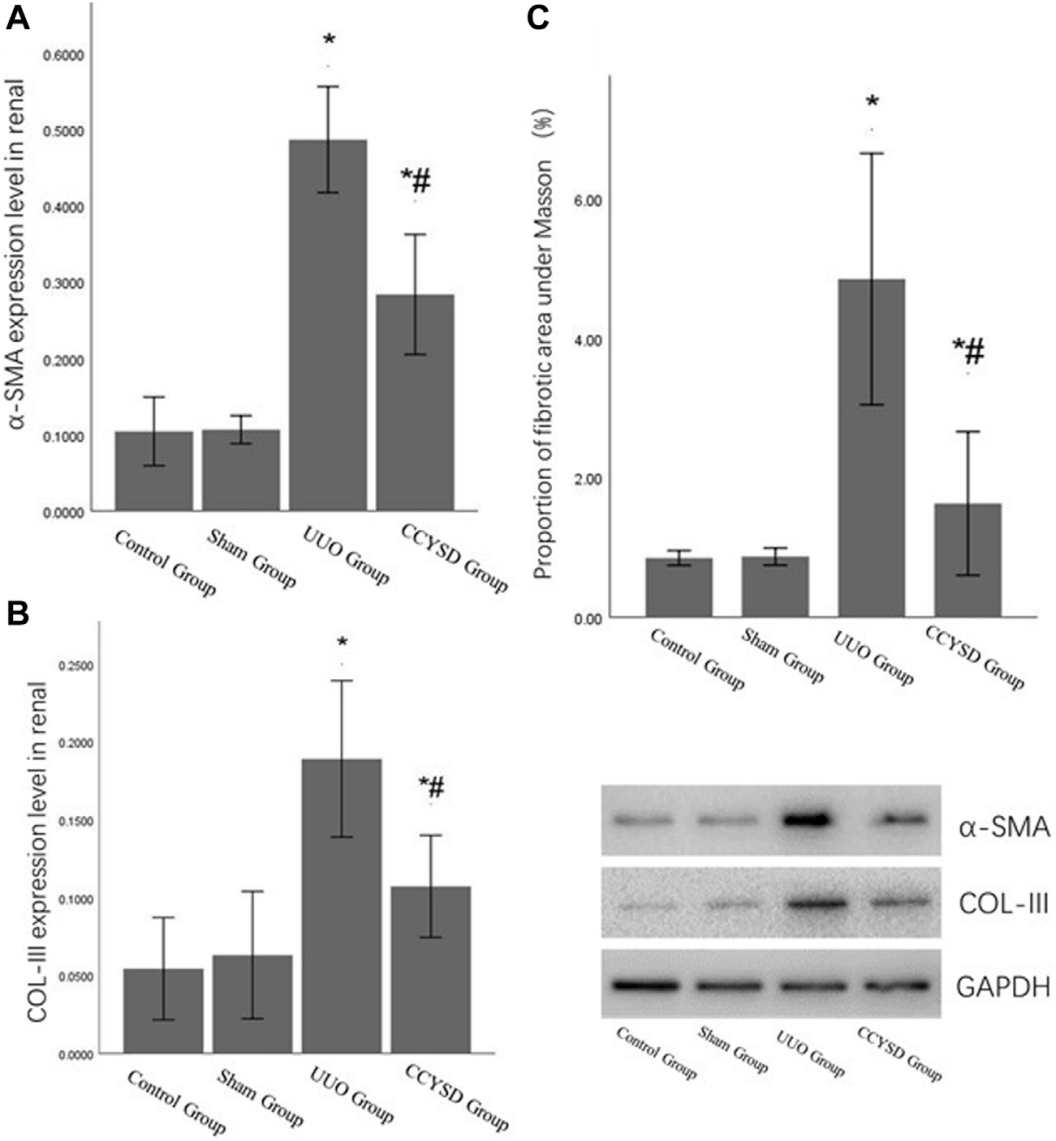
TIF levels in each group. **(A)**: α-SMA relative expression level in renal tissue; **(B)**: col-III relative expression level in renal tissue; **(C)**: the area of TIF with Masson staining; *compared with the sham group, *p* < 0.05; and ^#^compared with the UUO group, *p* < 0.05.

To evaluate the TIF, we detected fibrotic molecular marker (α-SMA and COL-III) expression levels and calculated the area of TIF with Masson staining. Compared with the control group, the expression levels of α-SMA and Col-III and the area of TIF in UUO renal tissues were significantly higher (*p <* 0.05). However, compared with the UUO group, the administration of CCYSD significantly meliorated fibrosis (*p <* 0.05) by lowering the expression levels of α-SMA and COL-III and shrinking the area of fibrosis (*p <* 0.05) (Figure 7).

### Oxidative Stress Level in the Body

In order to evaluate the levels of oxidative stress in rats, we invalidated the expression changes of MDA, SOD, and GSH in serum. It was found that compared with the sham group, the serum MDA level in the UUO group was significantly increased, while the levels of SOD and GSH were markedly decreased (*p* < 0.05) (Table 1). This kind of fluctuation from serum largely reflected the oxidative stress injury levels in kidney tissues (Hruska et al., 2017; Nordholm et al., 2018). However, CCYSD treatment significantly reversed these alterations in UUO rats, which indicates the increase in SOD and GSH and decrease in MDA (*p* < 0.05). All these findings suggested that CCYSD administration significantly attenuated the oxidative stress injury of CKD, which was consistent with the prediction results of network pharmacology analysis given above. Oxidative stress injury is likely to be the crucial target of CCYSD in treating CKD.

### Autophagy Level in Renal Tissue

**FIGURE 8:**
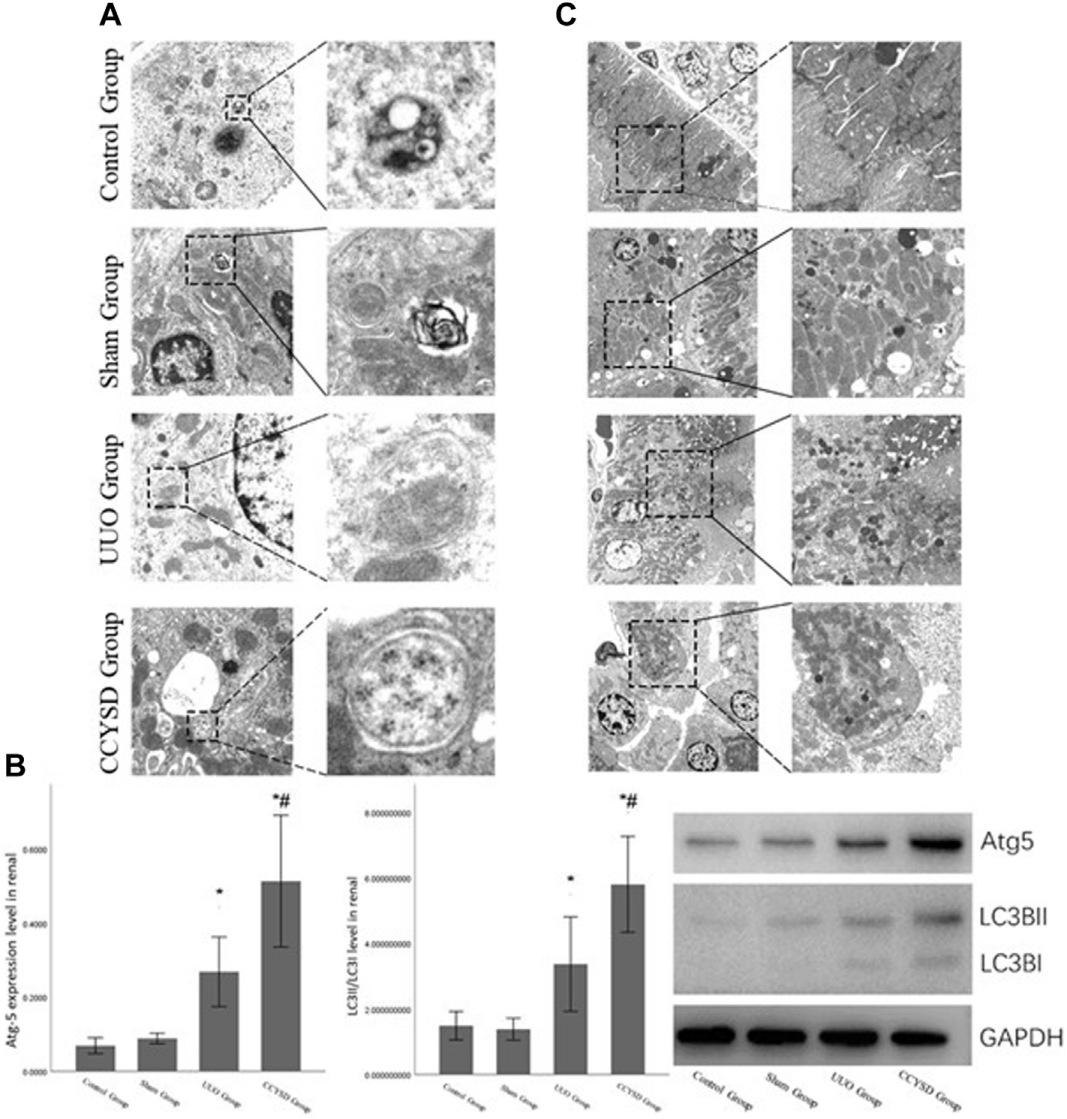
Autophagy and mitochondrial damage in the renal tissue. **(A)**: autophagosomes and autophagy-lysosomes in the renal tubular epithelial cells of each group under TEM; **(B)**: degree of mitochondrial injury in renal tubular epithelial cells in each group under TEM; **(C)**:the level of Atg5 and LC3II/LC3I ratio in renal tissue; *compared with the sham group, *p* < 0.05; and ^#^compared with the UUO group, *p* < 0.05).

To examine the effects of CCYSD on autophagy in renal tissue, the number and morphology of autophagosomes and autophagy-lysosomes were determined by transmission electron microscopy (TEM) (Figure 8A). A small amount of autophagosomes existed in normal renal tissues to maintain the circulation of substances in cells. On the contrary, the number of autophagosomes and autophagy-lysosomes was dramatically increased in renal tubular epithelial cells from the UUO group and further increased in the CCYSD group. Meanwhile, the level of Atg5 and LC3II/LC3I ratio in renal tissues verified the findings we discovered under TEM. The distinct autophagy was induced in renal tissues in the UUO group, and the activity of autophagy can be largely enhanced after the intervention of CCYSD (*p* < 0.05) (Figure 8C).

In addition, we also observed significant differences in the degree of mitochondrial damage in the renal tubule epithelial cells of each group under TEM. We found that severe mitochondrial damage observed in the renal tubular epithelial cells of the UUO group was relieved by treatment with CCYSD (Figure 8B). At the same time, we also discovered many subcellular structures such as mitochondria in some autophagosomes in CCYSD groups. Therefore, we speculated that the mitochondrial protective effect of CCYSD might be related to the activation of autophagy.

## Discussion

In recent years, as the prevalence of chronic diseases such as diabetes and hypertension increased with each passing year, chronic kidney disease secondary to that mentioned above has increased rapidly (Webster et al., 2017), which provokes enormous burdens on the public healthcare system around the world. Although growing importance has been attached to the prevention and treatment of CKD, a majority of patients will eventually receive dialysis or kidney transplantation because current treatment can only delay the progression of CKD to a certain extent. Abundant doctors are trying to apply Chinese medicine to the treatment of CKD, and massive research achievements have been obtained from clinical observation.

CCYSD, the renowned traditional Chinese herbal decoction, has been proven to be therapeutically effective and widely used in treating CKD for more than 10 years. What we had proved before was not only the clinical curative effect but also the potential mechanisms of CCYSD in treating CKD (Zhang, 2016; Mo et al., 2019a; Mo et al., 2019b). This study aims to detect the mechanism of CCYSD in treating CKD based on network pharmacology analysis, providing a supplement therapy strategy of TCM for CKD, and to further explore the specific mechanisms of CCYSD in treating CKD coupled with subsequent experimental validation. The network pharmacology systematically detected that the active ingredients such as cordycepin, quercetin, luteolin, and kaempferol play an important role in the treatment of CKD. 114 kinds of cellular functional activities and 112 related cellular signaling pathways were identified by GO and KEGG enrichment analysis mainly including apoptosis, autophagy, ubiquitin protein ligase system, protein phosphorylation, G protein-coupled receptor activation and serine/threonine kinase system, purine receptor family, regulation and control of nuclear transcription factor, hypoxia-inducing factor, inflammation, cell cycle regulation, hemodynamic regulation, and vascular endothelial cell injury, as well as a variety of cell functions and signaling pathways.

Notably, several signaling pathways and cell functions are closely related to autophagy, which were directly involved in autophagy regulation appearing in the drug regulatory network of CCYSD against CKD. Due to the vital role of autophagy regulation in CCYSD found through network pharmacological analysis, we carried out further exploration and verification in subsequent *in vivo* rats UUO model validation. Previous studies have confirmed that damaged mitochondria are the main sources of ROS in cells and induce oxidative stress damage in tissues (Shen et al., 2018). Autophagy can specifically degrade damaged mitochondria in cells and avoid the massive release of ROS when mitochondria rupture in order to reduce the oxidative stress damage caused by it. In this study, we found that tubulointerstitial fibrosis is directly related to oxidative stress injury, and CCYSD can delay this kind of fibrosis and significantly reduce oxidative stress injury *in vivo*. Transmission electron microscopy further showed that CCYSD can activate autophagy in epithelial cells and decrease mitochondrial damage. All these findings demonstrated that mitochondrial damage in the process of TIF can compensatively activate autophagy and, thus, play a self-protective role to some extent. Multiple bioactive components in CCYSD activate autophagy to delay TIF and treat CKD by degrading damaged mitochondria and ameliorating oxidative stress injury of tissues. Nevertheless, there were some limitations in the study. First, we only focused on the top 30 compounds and targets in the network pharmacology analysis, while ignoring these ranking after 30, which may attribute to a slight deviation of the results. Second, the validation of potential targets and signaling pathways is limited. Other predicted important targets and pathways not mentioned above require further experimental verification in the coming future.

## Conclusion

In summary, the pharmacological mechanism of CCYSD on chronic kidney disease may be mainly related to its autophagy activation. CCYSD can promote orderly degradation of damaged mitochondria and avoid mitochondrial rupture and ROS release by activating autophagy, thereby delaying the progression of TIF and CKD. However, 114 kinds of cellular functional activities and 112 related cellular signaling pathways were involved in this network pharmacological analysis. Except for the autophagy and oxidative stress injury, the pharmacological mechanism of CCYSD against CKD may also relate to inflammatory injury, cell cycle regulation, apoptosis, and other mechanisms. We only chose to verify the crucial role of the autophagy activity in the treatment of CKD with CCYSD, while other predicted vital targets and signaling pathways require further experimental verification in the future. Therefore, the secret of “multi-ingredients, multitargets, and multi-pathways mode” in the treatment of CCYSD against CKD needs further exploration.
